# An extensive investigation on human risk associated with PAHs in fish and sediment in Bushehr, Northern of Persian Gulf

**DOI:** 10.1038/s41598-024-61197-x

**Published:** 2024-05-08

**Authors:** Ghafour Nourian, Neamat Jaafarzadeh Haghighi Fard, Abdul Rahim Pazira, Esmaeil Kohgardi

**Affiliations:** 1 Department of Environmental, Bushehr Branch, Islamic Azad University, Bushehr, Iran; 2 Environmental Technologies Research Center, Medical Basic Sciences Research Institute, Ahvaz Jundishapur University of Medical Sciences, Ahvaz, Iran

**Keywords:** Polycyclic aromatic hydrocarbons, Human risk assessment, Sediment quality, Fish, ILCR, Ecology, Environmental sciences, Risk factors

## Abstract

Here, a comprehensive study was designed to estimate the human risk assessment attributed to exposure of polycyclic aromatic hydrocarbons (PAHs)in sediment and fish in most polluted shore area in north of Persian Gulf. To this end, a total of 20 sediment and inhabitual Fish, as one of most commercial fish, samples were randomly collected from 20 different stations along Bushehr Province coastline. The 16 different components of PAHs were extracted from sediment and edible parts of inhabitual fish and measured with high-performance liquid chromatography (HPLC) and gas chromatography (GC), respectively. In addition, dietary daily intake (DDI) values of PAHs via ingestion Indian halibut and the incremental lifetime cancer risk (ILCR) attributed to human exposure to sediments PAHs via (a) inhalation, (b) ingestion, and (c) dermal contact for two groups of ages: children (1–11 years) and adults (18–70 years) were estimated. The results indicated that all individual PAHs except for Benzo(b)flouranthene (BbF) and Benzo(ghi) perylene (BgP) were detected in different sediment sample throughout the study area with average concentration between 2.275 ± 4.993 mg.kg^–1^ dw. Furthermore, Naphthalene (Nap) with highest average concentration of 3.906 ± 3.039 mg.kg^–1^ dw was measured at the Indian halibut. In addition, the human risk analysis indicated that excess cancer risk (ECR) attributed to PAHs in sediment and fish in Asaluyeh with high industrial activities on oil and derivatives were higher the value recommended by USEPA (10^−6^). Therefore, a comprehensive analysis on spatial distribution and human risk assessment of PAHs in sediment and fish can improve the awareness on environmental threat in order to aid authorities and decision maker to find a sustainable solution.

## Introduction

The human and environmental concerns attributed to emission of toxic, persistent and non-biodegradable elements into biotic and abiotic matrices are of outstanding importance for sustainable and health future^1^. These elements can accumulate in organisms and microorganisms and even food chain and increase the levels of potential toxicity^2^. Polycyclic aromatic hydrocarbons (PAHs) with characteristics including semi-volatile, carcinogenicity, mutagenicity, and teratogenicity, bioaccumulation and long distance migration are a class of concerning contaminants, causing human and ecological risk^3^. PAHs with one or more benzene rings in their chemical structure are difficult to break for microorganisms; these persistent elements have long existed in different media of environments including air, water, soil and living organisms^4,5^. The PAHs are produced via both natural factors and anthropogenic activities^4^. The biodegradation, leaking, forest fires, eruptions from volcanoes and microbial production of subterranean fossil fuels are the major natural sources of PAHs^6^. In addition, the most important anthropogenic source of PAHs are industrial activities, combustion of fossil fuels and, automobile exhausts^7^. Given the thermodynamic characteristics, PAHs are generally divided into three categories: (1) pyrogenic (emitted from incomplete combustion and pyrolysis of organic material), (2) petrogenic (comes from crude oil and oil spills), (3) biogenic/diagenetic (released from slow transformation of organic matter in sediments)^1^. It is believed that rivers and sediments are the main host for accumulation of PAHs, especially, petrogenic ones^8^. The organic and inorganic pollutants enter the rivers and sediments via polluted wastewater, stormwater and effluent discharge. The PAHs are adsorbed on particles in sediments; these can be resuspended and rereleased into water sources^9^. In addition, toxic contaminants such as PAHs is bioacumulated in aquatic species such as fish and mussels^10^. As fish are one main meal of people and serve as food, it can contribute to significant health risks for humans^11^. As a consequence of rapid industrialization and urbanization, a huge amount of PAHs may be discharged into aquatic environments; they can deposit into environment, accumulate in biotic organism used for food chain and consequently damage the human nerves, respiratory, circulatory system^8,12^. Therefore, given the possible dangers imposed via entrance of PAHs and other toxic contaminant into sediment, water bodies and biotic organisms, a comprehensive data collection on sediment quality and bioaccumulation of contaminants in fish can aid deeper understanding of ecological and human risk assessment in the area^13,14^. Furthermore, a comprehensive and intense monitoring and assessment of spatial distribution of PAHs in sediments and fish can aid the decision maker to find a sustainable solution^15^. In this regards, many studies have focused on ecological and human risk of PAHs in sediments and foods^1,9,16–18^. For instance, Balram Ambade and et al.^16^ investigated the toxicity of PAHs in sediment in Damodar River Basin, India. The results indicated that concentration of 4-ring PAHs such as Fluoranthene (Flur), Pyrene (Pye), Benzo(a)Anthracene (BaA), and Chrysene (Chry) in the sediments were 43 ± 41 ng.g^–1^, 32 ± 29 ng.g^–1^, 52 ± 50 ng.g^–1^, and 83 ± 105 ng.g^–1^, respectively^16^. In addition, Xiaoqin Lin and et al.^18^ surveyed the human risk assessment of marine organisms in Shenzhen coastal waters in China. The authors reported that the individual lifetime cancer risk levels associated with seafood consumption in this area were ranged from 1.10 × 10^–5^ to 1.52 × 10^–5^, indicating the potential cancer risk for human which need special attention ^18^. Therefore, the present study was developed to comprehensively estimate the human risk assessment attributed to exposure to sediment and fish in one of most polluted area in Iran and even the world, where the industrial activities and oil refinery discharge is a serious threat for the environment.

## Methods and materials

### Study area description and sampling

**Figure 1:**
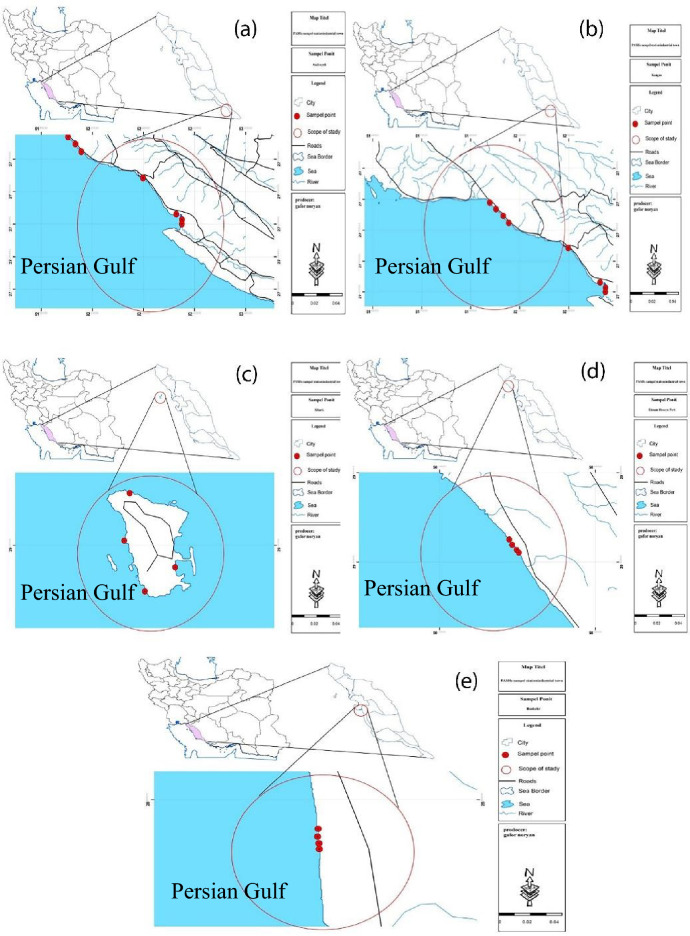
Case study location ((**a**) Assaluyeh, (**b**) Kangan, (**c**) Khark, (**d**) Emam Hasan, and (**e**) Bushehr) (Figure was prepared by Arc GIS version 10.3 (https://www.esri.com/en-us/home).

A comprehensive study on the sediment and Indian halibut samples was performed in order to determine the PAHs pollution in Bushehr province. To this end, five cities and island with high severity in oil pollution and industrial activities (Petrochemical and gas industries) throughout Busher Province, located at southeast of Iran were selected. Generally, a total of 20 samples were randomly collected from 20 different stations along Bushehr Province coastline including Asaluyeh, Kangan, Khark, Emam Hasan, and Bushehr from January 2019 to February 2019 (Fig. 1). The geographical location of selected stations is shown in Fig. 1. The sediment samples were withdrawn based on five-point sampling approach with a length of 10 m. Briefly, at each station, five sediment samples at four corners and center with a depth of 5 cm were collected. In general, 3 kg sediment samples were immediately placed in a pre-cleaned glass container with polytetrafluoroethylene screw caps and immediately transported to the laboratory.

In case of fish samples, 20 inhabitual Fish samples as one of most commercial fish along the Persian Gulf coastline were withdrawn by a trawl net in different cities and island mentioned earlier. The fish samples were immediately kept at cool box and transferred to laboratory for further analysis. Then, the fish samples were washed with distilled water and prepared to extract PAHs from their edible parts. Then, the edible parts of fish samples were put in the oven at 80 °C for 18 h. The dried samples were powdered for PAHs extraction and further analysis^19^.

### PAHs extraction from sediment and fish samples

Extraction of PAHs from sediment samples was carried out according to the following procedure: 250 mL dichloromethane-n-hexane (1:1) was added to 10 g of each freeze-dried sediment and then the mixture was placed in soxhlet for 8 h. The residual was condensed to 15 mL by a rotary evaporator. Active copper (2–3 g) was added to the residual for eliminating sulfur, and so the mixture was filtered to 24 h. The residual was passed from a column comprising 10 g silica, 10 g active alumina, and 2 g anhydrous sodium sulfate. The mixture was concentrated to 5 ml again and was placed in scaled vials. Once the solvent was evaporated completely, 1 mL acetonitrile was added to the sample for high-performance liquid chromatography (HPLC) injection^20^. In case of PAHs in fish samples, 5 g dried edible part of Indian halibut was carefully mixed with 5 mL KOH (50%) and 75 mL methanol. The mixture was placed in soxhlet for 4 h. After transferring the liquid phase to the separator funnel, 100 mL n-hexane was added and vigorously shaken for 3 min. The methanol- KOH solution was evacuated and then hexane phase was also washed with 50 mL methanol–water solution (8:1) and 50 ml water. The samples were passed from the column containing 15 g silica, 10 g active alumina, and 1 g anhydrous sodium sulfate. 1 mL n-hexane was added to each sample to prepare for further analysis and Gas Chromatography (GC) analysis.

### Analytical analysis

The measurement of different components of PAHs in the samples were performed by a gas chromatography–mass spectrometry (GC 6890, AGILENT, MS 5973N, Mode EI, detector:MS).

Generally, the concentrations 16 different aromatic compounds: Acenaphthylene, Naphthalene, Acenaphthene, Phenanthrene, Anthracene, Fluoranthene, Fluorene, Pyrene, Chrysene, Benzo(b)fluoranthene, Benzo(k)fluoranthene, Benzo(a)anthracene, Benzo[a]pyrene, Indeno(1,2,3,cd)pyrene, Benzo(g.h.i)perylene, Dibenz[a,h]anthracene were analyzed according to EPA method 3500C^21^ and National standard of Iran (19,238)^22^.

### Risk assessment

The dietary daily intake (DDI) values of PAHs via ingestion Indian halibut were estimated by assumption an average weight of 70 kg for adults using Eq. (1)^11^,

1 $$ {\text{DDI }}\left( {{\text{mg}}/{\text{day}}} \right) \, = {\text{ Ci }} \times {\text{ IR}}, $$

where Ci is PAHs concentration as mg/kg and IFR denotes ingestion rate (kg/day). The toxicity and carcinogenity of the high molecular weight PAHs compared to BaP were determined by toxic equivalent quotient (TEQ). This quotient is related to the valence of any congener for create modification in DNA of human. The BaP carcinogenic equivalent (TEQ) for the individual PAHs in sediment and fish samples was calculated by Eq. (2)^19^.

2 $$ {\text{TEQ }} = \, \sum {\left( {{\text{TEFn }} \times {\text{ Ci}}} \right)} , $$

where Ci = concentration of the individual PAHs in the sampling sediments and Indian halibuts (mg.kg^–1^ dw), and TEFn is toxic equivalence factor for individual PAHs.

The incremental lifetime cancer risk (ILCR) evaluates exposure health risk from PAHs according to USEPA standard. The major pathways for human exposure to sediments PAHs include (a) inhalation, (b) ingestion, and (c) dermal contact, with the age categories of children (1–11 years) and adults (18–70 years). The following Eqs. (3), (4), (5) were applied to evaluate the incremental lifetime cancer risk (ILCR) from PAHs in the sediments^23^.

3 $${\text{ILCR}}\left({\text{Ingestion}}\right)= \frac{\sum TEQ . \left\{{CSF}_{ingestion} ({BW/70)}^\frac{1}{3}\right\}. ED . EF . {IR}_{ingestion}}{BW . AT . {10}^{6}},$$

4 $${\text{ILCR}}\left({\text{Inhalation}}\right)= \frac{\sum TEQ . \left\{{CSF}_{inhalation} ({BW/70)}^\frac{1}{3}\right\}. ED . EF . {IR}_{inhalation}}{BW . AT . BEF},$$

5 $${\text{ILCR}}\left(\mathrm{Dermal contact}\right)= \frac{\sum TEQ . \left\{{CSF}_{\mathrm{Dermal \,\,contact}} ({BW/70)}^\frac{1}{3}\right\}. ABS . AF . SA . EF . ED}{BW . AT . {10}^{6}}.$$

In addition, the excess cancer risk of PAHs due to ingestion Indian halibut was estimated using Eq. (6)^11^.

6 $$ {\text{Excess Cancer Risk }}\left( {{\text{ECR}}} \right) \, = \, \Sigma {\text{Q }} \times {\text{ B }}\left( {\text{A}} \right){\text{ Pteq }} \times {\text{ IFR }} \times {\text{ ED}}/\left( {{\text{BW }} \times {\text{ ATn}}} \right). $$

Human risk model parameters in the sediment and Indian halibut samples are presented in Tables1 and 2, respectively.

**Table 1:** Health risk model components at the sediment samples.

Components	Unit	Value
Carcinogenic slope factor for ingestion (CSF_Ingestion_)	mg kg^–1^ day^–1^	7.3
Carcinogenic slope factor for dermal (CSF_Dermal_)	mg kg^–1^ day^–1^	25
Carcinogenic slope factor for inhalation (CSF_Inhalation_)	mg kg^–1^ day^–1^	3.85
Body weight (BW)	Kg	Child: 15, adult: 61.5
Exposure frequency of the (EF)	day. a^–1^	180
Exposure duration (ED)	Years	Child: 6, adult: 24
Inhalation rate (IR_Inhalation_)	m^3^ day^–1^	Child: 10, adult: 20
Dust ingestion rate (IR_Ingestion_)	mg day^–1^	Child: 200, adult: 100
Dermal exposure area (SA)	cm^2^	Child: 2800, adult: 5700
Dermal adherence factor (AF)	mg cm^–2^	Child: 0.2, adult: 0.07
Dermal adsorption fraction (ABS)		0.13
Averaging life span (AT)	Days	25,550
Particle emission factor (PEF)	m^3^ kg^–1^	1.36 × 10^9^

**Table 2:** Health risk model parameters in the Indian halibut samples.

Parameters	Unit	Value
Concentration of each congener (Ci)	mg.kg^–1^	–
Fish ingestion rate (IFR)	Kg.capita^–1^.day^–1^	0.0548
Carcinogenic potency of Benzo[a]Pyrene (Q)	Mg.kg^–1^d^–1^	7.3
Exposure duration (ED)	Years	30
Adult body weight (BW)	kg	70
Average lifespan (ATn)	Days	8760

### Data analysis

The SPSS-23 software was applied to the statistical analyses of results. The results were presented as the average ± standard deviation (SD). The correlation between data was tested with Pearson’s correlation coefficient and analysis of variance (ANOVA). The level of P < 0.05 was significant.

### Ethics declarations

We declare that this study was conducted in compliance with the ARRIVE (animal research: reporting of in vivo experiments) guidelines, adhering to its core principles and guidelines. We have followed the necessary steps outlined in the ARRIVE guidelines to minimize suffering and ensure appropriate care and welfare for the experimental animals. We have provided detailed descriptions of the methods and procedures of the animal experiments, and have reported relevant statistical analyses and data comprehensively. The method of this study has been approved by the Ethics Committee of Bushehr Branch, Islamic Azad University, Bushehr, Iran, and the animal experiments were approved by the Ethics Committee of Bushehr Branch, Islamic Azad University, Bushehr, Iran (16-11-8458, 1400/02/05).

## Results and discussion

### PAHs concentration in sediments

The mean concentration of different compounds of PAHs in different sediment samples drawn from five cities in Bushehr province is summarized in Table 3.

**Table 3:** PAHs concentrations evaluated in the coastal sediments and Indian halibuts of Bushehr province.

Compounds	Sediments			Indian halibuts		
	TEF	Mean (mg.kg^–1^ dw)	Std.De	TEF	Mean (mg.kg^–1^ dw)	Std.De
Naphthalene (Nap)	–	0.518	1.598	0.001	3.906	3.039
Acenaphthylene (Acy)	–	1.705	4.291	0.001	2.282	3.139
Acenaphthene (Ace)	–	2.275	4.993	0.001	1.224	1.471
Flourene (Fl)	–	0.334	0.624	0.001	1.074	0.909
Phenanthrene (Phe)	–	0.006	0.016	0.001	0.052	0.053
Anthracene (Ant)	–	0.435	0.997	0.01	0.532	0.832
Flouranthene (Flu)	–	0.005	0.015	0.001	0.270	0.603
Pyrene (Pyr)	–	0.309	0.924	0.001	1.034	0.969
Benzo(a) anthracene (BaA)	0.1	0.011	0.029	0.1	0.122	0.167
Chrysene (Chry)	0.001	0.013	0.034	0.01	0.066	0.121
Benzo(b)flouranthene (BbF)	0.1	ND	00.00	1	0.040	0.055
Benzo(k)flourathene (BkF)	0.01	0.036	0.060	0.1	0.178	0.245
Benzo(a) pyrene (BaP)	1	0.004	0.012	0.1	0.054	0.099
Dibezo(ah) anthracene (DbA)	1	0.751	2.242	1	1.388	1.538
Benzo(ghi) perylene (BgP)	–	ND	00.00	0.01	0.530	1.005
Indeno (1,2,3-cd) pyrene (IdP)	0.1	0.482	1.032	0.1	2.044	1.426
ƩPAHs	–	6.894	1.4301	–	14.796	7.476
ƩTEQ	–	0.804	2.338	–	1.692	1.533

*ND* not detection, *Std*.*De* standard deviation, *dw* dry weight.

**Figure 2:**
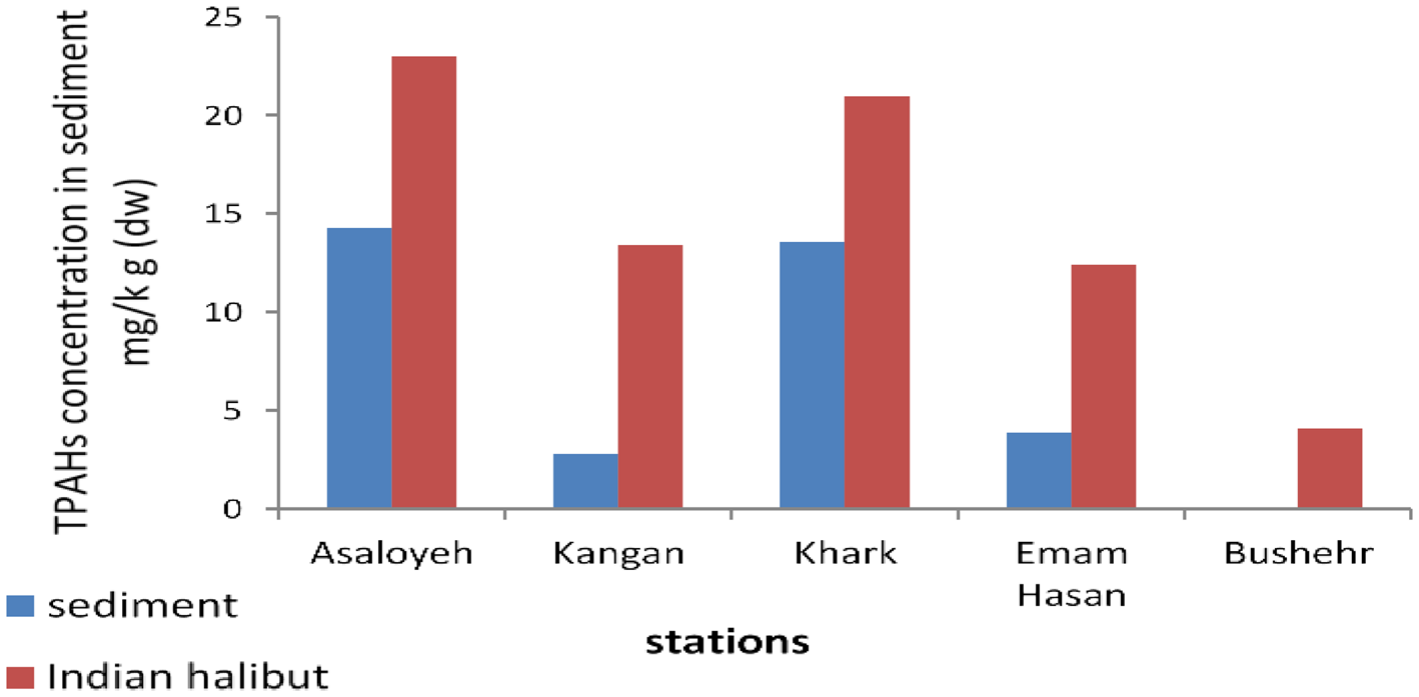
The mean concentration of PAHs in different sampling stations.

As summarized in Table 3, the individual PAHs except for Benzo(b)flouranthene (BbF) and Benzo(ghi) perylene (BgP) were detected in different sediment stations of study area. In addition, Acenaphthene was measured with highest average concentration of 2.275 ± 4.993 mg.kg^–1^ dw at the sediment. The lowest mean concentration of PAHs in the sediment belonged to Benzo(a) pyrene (BaP) 0.004 ± 0.012 mg.kg^–1^ dw. The average PAHs concentrations in the sediment samples of Asaluyeh, Kangan, Khark, Emam Hasan, and Bushehr stations are illustrated in Fig. 2.

As illustrated in Fig. 2, the average total PAHs concentration in Asaluyeh, Kangan, Khark, and Emam Hasan stations were 14.267, 2.777, 13.562, 3.865 mg.kg^–1^ and ND, respectively. Of interesting, no PAH components were detected in Bushehr station. The most possible reason for differences in PAHs concentration in different sampling stations are attributed to type and amounts of pollutants derived from industrial activities. In addition, factors such as organic carbon content, structure, diameter of sediment particles, and water solubility are effective in the distribution and abundance of PAHs in sediments^24^. Ali Ranjbar Jafarabadi and et al.^25^ surveyed the PAHs concentration in three islands (Qeshm, Hengam, Hormoz) with dense human activities; the authors reported that PAHs in reef surface sediments were in range of 274 to 1098 ng. g^–1^ dw^25^. In addition, Gui Wang and et al.^4^ surveyed the PAHs concentrations in surface sediments from Dingzi Bay, China. The authors reported that PAHs in sediment samples were ranged from 71.38 to 163.28 ng⋅g^–1^^4^.

### PAHs concentration in Indian halibut fish

Table 3 summarizes the mean concentration of different individual PAHs in edible parts of Indian halibut samples drawn from different stations. As summarized in Table 3, the individual PAHs were detected in different edible parts of Indian halibut samples. In addition, Naphthalene (Nap) was measured with highest average concentration of 3.906 ± 3.039 mg.kg^–1^ dw at the Indian halibut. The lowest mean concentration of PAHs in the Indian halibut belonged to Benzo(a) pyrene (BaP) 0.040 ± 0.055 mg.kg dw. The average PAHs concentrations in the Indian halibut samples of Asaluyeh, Kangan, Khark, Emam Hasan, and Bushehr stations are illustrated in Fig. 2. As illustrated in Fig. 2, the average total PAHs concentration in Asaluyeh, Kangan, Khark, Emam Hasan, and Bushehr stations were 22.999, 13.400, 20.977, 12.400 and 4.260 mg.kg^–1^ dw, respectively. The ƩPAH concentration was highest at Asaluyeh area, but was not significantly difference (p > 0.05) among the samples of PAHs. Furthermore, PAHs concentrations in the edible part of fishes were higher than those in the sediments, suggesting the ability of PAHs accumulation by the fishes. Due to the presence of fat tissues of living organisms, the pollutants are absorbed effectively in their tissues. On the other hand, hydrocarbon pollutants present in the sediments are decomposed in various ways such as optical photosynthesis and microbial processes^26,27^. Hongliang Zhang and et al.^28^ surveyed the PAHs in four common wild marine fish in Zhoushan Archipelago, East China Sea. The authors reported that PAHs concentration in edible muscles of fishes were 34.7–108 ng.g^–1^ wet weight and Four-ring and six-ring PAH congeners constitute the most and least percentages of the total PAHs, respectively^28^. Xiaoqin Lin and et al. surveyed the PAHs concentration in marine organisms in Shenzhen coastal waters of China and the results indicated that benzo[*a*]pyrene with concentration in range of non-detectable to 11.21 ng. g^–1^ dw was detected in most species^18^.

### Human risk assessment

#### Human risk assessment attributed to exposure to sediments containing PAHs

Table 4 summarizes the ILCR attributed to exposure to PAHs in sediments via ingestion, inhalation and dermal. ILCR for two groups of ages including child and adults were calculated in the present study (See Table 4). As summarized in Table 4, the average cancer risk for adult living in Asaluyeh, Kangan, Khark and Emam Hasan were 1.00 × 10^–5^, 1.93 × 10^–7^, 1.10 × 10^–5^ and 1.82 × 10^–7^, respectively. In addition, the average cancer risk for child living in Asaluyeh, Kangan, Khark and Emam Hasan were 1.04 × 10^–5^, 1.11 × 10^–7^, 1.14 × 10^–5^, and 1.89 × 10^–7^, respectively. Generally, the order of average cancer risk in four sampling stations for adults were as follows: Khark > Asaluyeh > Kangan > Emam Hasan. In addition, the order of average cancer risk in four sampling stations for child were as follows: Khark > Asaluyeh > Emam Hasan > Kangan. It is important to note that and as earlier mentioned, no PAH components were detected in Bushehr station. Therefore, ILCR was not reported in Table 4.

**Table 4:** Potential cancer risk values of PAHs in sediments for children and adults.

Stations		Child				Adult			
		ILCR ingestion	ILCR inhalation	ILCR dermal contact	Cancer risk	ILCR ingestion	ILCR inhalation	ILCR dermal contact	Cancer risk
Asaluyeh	1	2.70 × 10^–7^	5.24 × 10^–12^	3.37 × 10^–7^	6.07 × 10^–7^	2.11 × 10^–7^	1.63 × 10^–11^	3.75 × 10^–7^	5.86 × 10^–7^
	2	3.72 × 10^–7^	7.21 × 10^–12^	4.63 × 10^–7^	8.35 × 10^–7^	2.90 × 10^–7^	2.25 × 10^–11^	5.15 × 10^–7^	8.06 × 10^–7^
	3	2.21 × 10^–8^	4.29 × 10^–13^	2.76 × 10^–8^	4.97 × 10^–8^	1.72 × 10^–8^	1.34 × 10^–12^	3.07 × 10^–8^	4.79 × 10^–8^
	4	1.78 × 10^–5^	3.46 × 10^–10^	2.22 × 10^–5^	4.01 × 10^–5^	1.39 × 10^–5^	1.08 × 10^–9^	2.47 × 10^–5^	3.87 × 10^–5^
	Ave	4.63 × 10^–6^	8.98 × 10^–11^	5.77 × 10^–6^	1.04 × 10^–5^	3.61 × 10^–6^	2.80 × 10^–10^	6.42 × 10^–6^	1.00 × 10^–5^
Kangan	1	8.65 × 10^–8^	1.67 × 10^–12^	1.07 × 10^–7^	1.94 × 10^–7^	6.75 × 10^–8^	5.23 × 10^–12^	1.19 × 10^–7^	1.87 × 10^–7^
	2	1.32 × 10^–7^	2.57 × 10^–12^	1.65 × 10^–7^	2.98 × 10^–7^	1.03 × 10^–7^	8.04 × 10^–12^	1.84 × 10^–7^	2.87 × 10^–7^
	3	1.37 × 10^–7^	2.67 × 10^–12^	1.71 × 10^–7^	3.09 × 10^–7^	1.07 × 10^–7^	8.34 × 10^–12^	1.91 × 10^–7^	2.98 × 10^–7^
	4	2.95 × 10^–10^	5.72 × 10^–15^	3.68 × 10^–10^	6.63 × 10^–10^	2.30 × 10^–10^	1.78 × 10^–14^	4.09 × 10^–10^	6.39 × 10^–10^
	Ave	8.93 × 10^–8^	1.73 × 10^–12^	1.11 × 10^–7^	2.00 × 10^–7^	6.97 × 10^–8^	5.41 × 10^–12^	1.23 × 10^–7^	1.93 × 10^–7^
Khark	1	3.12 × 10^–7^	6.05 × 10^–12^	3.89 × 10^–7^	7.01 × 10^–7^	2.43 × 10^–7^	1.89 × 10^–11^	4.33 × 10^–7^	6.77 × 10^–7^
	2	3.47 × 10^–7^	6.73 × 10^–12^	4.32 × 10^–7^	7.79 × 10^–7^	2.71 × 10^–7^	2.10 × 10^–11^	4.81 × 10^–7^	7.54 × 10^–7^
	3	2.21 × 10^–8^	4.29 × 10^–13^	2.76 × 10^–8^	4.97 × 10^–8^	1.72 × 10^–8^	1.34 × 10^–12^	3.07 × 10^–8^	4.79 × 10^–8^
	4	1.96 × 10^–5^	3.81 × 10^–13^	2.45 × 10^–5^	4.42 × 10^–5^	1.53 × 10^–5^	1.19 × 10^–9^	2.72 × 10^–5^	4.26 × 10^–5^
	Ave	5.09 × 10^–6^	9.87 × 10^–11^	6.34 × 10^–6^	1.14 × 10^–5^	3.97 × 10^–6^	3.08 × 10^–10^	7.05 × 10^–6^	1.10 × 10^–5^
Emam Hasan	1	0 ×	0	0	0	0	0	0	0
	2	2.48 × 10^–7^	4.81 × 10^–12^	3.09 × 10^–7^	5.58 × 10^–7^	1.93 × 10^–7^	1.50 × 10^–11^	3.44 × 10^–7^	5.38 × 10^–7^
	3	0	0	0	0	0	0	0	0
	4	8.85 × 10^–8^	1.71 × 10^–12^	1.10 × 10^–7^	1.98 × 10^–7^	6.91 × 10^–8^	5.36 × 10^–12^	1.22 × 10^–7^	1.91 × 10^–7^
	Ave	8.42 × 10^–8^	1.63 × 10^–12^	1.05 × 10^–7^	1.89 × 10^–7^	6.57 × 10^–8^	5.10 × 10^–12^	1.16 × 10^–7^	1.82 × 10^–7^

Ruqayah Ali Grmasha and et al. surveyed the human risk assessment attributed to exposure to PAHs in sediment in along Euphrates River. The results indicated that the calculated incremental lifetime cancer risk (ILCR) for adult and children were in the 10^–2^–10^–3^ range, which are higher than the values calculated in this study^1^. In addition, Yanan Chen and et al. reported that the ILCR for children and adult exposed to PAHs in farmland soil of Yinma River Basin, China were 1 × 10^–6^–1.36 × 10^–6^ and 1.40 × 10^–6^–4.22 × 10^–6^, respectively, which are comparable with the results presented in the current study^29^.

#### Human risk assessment attributed to ingestion inhabitual fish containing PAHs

Table 5 summarizes concentration (Ci), Dietary daily intake (DDI), Carcinogenic potencies (B(A)Pteq), and excess cancer risk (ECR) of different PAHs in Indian halibut samples withdrawn from five sampling stations in Bushehr province. As summarized in Table 5, no PAHs were detected in Indian halibut samples withdrawn from Bushehr; there is no human risk for ingestion the Indian halibut in this area. However, almost all different components of PAHs were detected in Indian halibut samples withdrawn from Asaluyeh, Kangan and Khark. While some PAHs component were not detected in Emam Hasan (See Table 5). In addition, as summarized in Table 5, the excess cancer risk (ECR) for Asaluyeh, Kangan, Khark and Emam Hasan for different components of PAHs in Indian halibut samples were 1.76 × 10^–9^–6.2 × 10^–6^, 5.28 × 10^–9^–5.96 × 10^–5^, 9.78 × 10^–10^–1.74 × 10^–5^, and 2.34 × 10^–9^–5.61 × 10^–6^, respectively. Therefore, the highest ECR were calculated in Kangan and Asaluyeh with high industrial activities on oil and derivatives. In the present research, the ECR values for every individual PAH except for DbA were less than threshold value of USEPA (10^−6^) (Table 5), but the collective values of ECR in Indian halibut samples in all studied areas exceeded the USEPA acceptable level^30^. Thus, high consumption of Indian halibut is the main and most probable reason for high potential cancer risk in the study area. Xiaoqin Lin and et al. surveyed the human health risk assessment attributed to consumption of marine organisms containing PAHs from Shenzhen coastal. The authors reported that the individual lifetime cancer risk associated with seafood consumption ranged from 1.10 × 10^–5^ to 1.52 × 10^–5^, indicating a potential cancer risk needed special attention. These results are in line with the results presented in the current study^18^. Chuchu Zhang and et al. investigated the PAHs concentration and attributed human risk assessment due to consumption of marine organisms from two fishing grounds, South Yellow Sea, China. The authors reported that the ECR from PAH-contaminated seafood consumption was slightly higher than the guideline value (10^–6^)^31^. Overall, the high ECR attributed to consumption of fish contain PAHs in Bushehr province with high industrial activities and oil and gas refinery is a great risk for people living in this area. Therefore, preventative measurements are required for lower the human risk assessment; the authorities must make the industries to meet the standard for discharge of effluent into aquatic environments.

**Table 5:** Concentration (Ci), dietary daily intake (DDI), carcinogenic potencies (B(A)Pteq), and excess cancer risk (ECR) of PAHs in Indian halibut samples.

PAH s	Concentration (Ci) (mg.kg^-1^)					DDI					B(A)Pteq					ECR				
	A	K	Kh	EH	B	A	K	Kh	EH	B	A	K	Kh	EH	B	A	K	Kh	EH	B
NaP	6.48	1.43	4.89	6.73	ND	3.55E-01	7.84*E–02	2.67E–01	3.68E–01	0	6.48E–03	1.43E–03	4.89E–03	6.73E–03	0	1.26E–07	2.79E–08	9.57E–08	1.31E–07	0
Acy	5.27	ND	6.14	ND	ND	2.88E–01	0	3.36E–01	0	0	5.27E–03	0	6.14E–03	0	0	1.03E–07	0	1.20E–07	0 b	0
Ace	1.60	3.56	0.96	ND	ND	8.79E–02	1.95E–01	5.26E–02	0	0	1.60E–03	3.56E–03	9.60E–04	0	0	3.13E–08	6.96E–08	1.87E–08	0	0
FL	1.32	0.48	2.39	1.18	ND	7.23E–02	2.63E–02	1.31E–01	6.46E–02	0	1.32E–03	4.80E–04	2.39E–03	1.18E–03	0	2.58E–08	9.39E–09	4.68E–08	2.30E–08	0
Phe	0.09	ND	0.05	0.12	ND	4.93E–02	0	2.74E–03	6.57E–03	0	9.00E–05	0	5.00E–05	1.20E–04	0	1.76E–09	0	9.78E–10	2.34E–09	0
Ant	1.98	0.21	ND	0.47	ND	1.08E–01	1.15E–02	0	2.57E–02	0	1.98E–02	2.10E–03	0	4.70E–03	0	3.87E–07	4.11E–08	0	9.19E–08	0
Flu	ND	ND	1.35	ND	ND	0	0	7.39E–02	0	0	0	0	1.35E–03	0	0	0	0	2.64E–08	0	0
Pyr	2.45	0.27	1.59	0.69	0.17	1.34E–01	1.47E–02	8.76E–02	3.78E–02	9.31E–03	2.45E–03	2.70E–04	1.59E–03	6.90E–04	1.70E–04	4.80E–08	5.28E–09	3.12E–08	1.35E–08	3.32E–09
BaA	ND	0.32	0.29	ND	ND	0	1.75E–02	1.58E–02	0	0	0	3.20E–02	2.90E–02	0	0	0	6.26E–07	5.67E–07	0	0
Chr	0.05	0.28	ND	ND	ND	2.74E–02	1.53E–02	0	0	0	5.00E–04	2.80E–03	0	0	0	9.78E–09	5.48E–08	0	0	0
BbF	0.09	0.11	ND	ND	ND	4.93E–02	6.02E–02	0	0	0	9.00E–02	1.10E–01	0	0	0	1.76E–06	2.15E–06	0	0	0
BkF	0.57	0.27	0.05	ND	ND	3.12E–02	1.47E–02	2.74E–03	0	0	5.57E–02	2.70E–2	5.00E–03	0	0	1.11E–06	5.28E–07	9.78E–08	0	0
BaP	ND	0.23	0.04	ND	ND	0	1.26E–02	2.19E–03	0	0	0	2.30E–02	4.00E–03	0	0	0	4.50E–07	7.82E–08	0	0
DbA	ND	3.05	0.89	ND	3.00	0	1.67E–01	4.90E–02	0	1.64E–01	0	3.05E00	8.90E0–1	0	3.00E00	0	5.96E–05	1.74E–05	0	5.87E–05
BgP	ND	ND	2.31	0.34	ND	0	0	1.27E–01	1.86E–02	0	0	0	2.31E–02	3.40E–03	0	0	0	4.53E–07	6.65E–08	0
IdP	3.08	3.18	ND	2.87	1.09	1.69E–01	1.74E–01	0	1.57E–01	5.97E–02	3.08E–01	3.18E–01	0	2.87E–01	1.09E–01	6.02E–06	6.22E–06	0	5.61E–06	0

*ND* not detection.

## Conclusion

The present study was developed to comprehensively investigate the human exposure assessment of PAHs for people living in one of most polluted area in the world due to industrial activities and oil refinery. To this end, different components of PAHs in sediments and fish samples in the north area of Persian Gulf were measured and the corresponding human risk assessment following ingestion, inhalation and dermal pathway were estimated. Overall, the human risk analysis and ECR attributed to PAHs in sediment and fish in Asaluyeh, as well-known most polluted area, were much higher than the value recommended by USEPA (10^−6^). Furthermore, a comprehensive and intense monitoring and assessment on spatial distribution of PAHs in sediments and fish can aid the authorities and decision maker to find a sustainable solution.

## Data Availability

All data generated or analysed during this study are included in this published article.
